# Endodontic Retreatment of a Mandibular First Premolar With Unusual Four Root Canal Morphology: A Case Report

**DOI:** 10.1155/crid/4489618

**Published:** 2026-10-07

**Authors:** Hsin Lan, Yi Chen Chiang, Chin Sung Chen, Yun Ting Liu

**Affiliations:** ^1^ Department of Endodontics Sijhih Cathay General Hospital New Taipei Taiwan cgh.org.tw; ^2^ Department of Endodontics Cathay General Hospital Taipei Taiwan cgh.org.tw; ^3^ Taipei Medical University School of Dentistry Taipei Taiwan

**Keywords:** cone-beam computed tomography, dental operating microscope, dentistry, premolar, radiography, root canal anatomy

## Abstract

**Introduction:**

Mandibular premolars exhibit complex root canal systems, making them highly challenging to manage endodontically. Canal configurations vary from one to five canals. Successful endodontic treatment requires a thorough knowledge of standard anatomy and its variations.

**Case Presentation:**

This case report presents the endodontic retreatment of a right mandibular first premolar with four separate root canals in a 36‐year‐old male. Initial clinical and radiographic examinations showed a complex root canal anatomy. With the aid of a dental operating microscope (DOM) and cone‐beam computed tomography (CBCT), a missed distolingual canal was successfully identified along with the mesiobuccal, mesiolingual, and distobuccal canals. The root canals were prepared, disinfected, and densely obturated.

**Conclusion:**

This report highlights the importance of detailed root canal exploration using advanced diagnostic tools like DOM and CBCT to ensure the successful management of rare anatomical variations in mandibular premolars.

## 1. Introduction

Successful endodontic treatment depends on complete cleaning and shaping via a combination of biomechanical instrumentation, chemical disinfection, dense obturation, and a hermetic coronal seal. Variations in root canal systems and undetected canals are frequently observed in endodontic flare‐ups and failures [1]. A previous study showed that the mandibular first premolar has the highest failure rate (11.45%) among all types of teeth after nonsurgical root canal treatment [2]. The complexity of the internal anatomy and morphology of mandibular first premolars contributes significantly to these high failure rates. The primary reason for treatment failure is the persistence of residual microorganisms inside the unmanaged spaces of the root canal system [3]. Therefore, it is crucial for clinicians to be thoroughly equipped with the knowledge of tooth anatomy and to be aware of the possibility of additional canals prior to and during endodontic treatment.

To locate extra root canals, the resolution of the optical field can be substantially improved by using a dental operating microscope (DOM). Furthermore, when two‐dimensional (2D) imaging does not provide sufficient information regarding the actual number of roots and canals, cone‐beam computed tomography (CBCT) can be utilized to accurately evaluate the root canal system [4].

## 2. Case Presentation

A 36‐year‐old male patient with no significant history of systemic diseases or allergies presented to the Department of Endodontics for the management of a fractured right lower tooth. The patient reported that the tooth had received root canal treatment 10 years prior and had remained asymptomatic for a long period.

Clinical examination revealed a crown fracture of Tooth 44, accompanied by a dislodged composite resin filling, evident secondary caries, and exposed gutta‐percha. The tooth was nontender to percussion and palpation. No significant mobility or deep periodontal pocket depths were noted upon probing.

Radiographic examination showed that Tooth 44 had been previously endodontically treated and exhibited a periapical lesion. It was also noted that the existing obturation material was not centered within the root outline, and two distinct periodontal ligament (PDL) spaces were observable along the mesial aspect. Based on these radiographic findings, the presence of multiple canals was strongly suspected (Figure 1a). According to the American Association of Endodontists (AAE) diagnostic criteria, the definitive pulpal and periapical diagnosis was previously treated and asymptomatic apical periodontitis.

**Figure 1:**
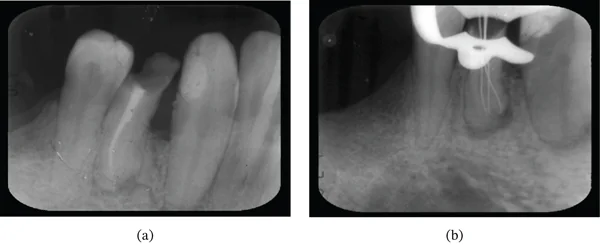
Radiographic evaluation of Tooth 44. (a) Preoperative periapical radiograph revealing an eccentric root canal filling relative to the root outline, along with two distinct periodontal ligament (PDL) spaces on the mesial aspect. (b) Working length radiograph demonstrating that the intracanal instruments deviate from the root center, indicating the presence of anatomical variations and missed canals.

At the first appointment, dental caries and softened dentin were completely removed, and an access opening was achieved under rubber dam isolation. The removal of the existing gutta‐percha was carried out using eucalyptus oil (Kemig d.o.o. Zagreb, Croatia), Gates Glidden drills (Maillefer, Ballagius, Switzerland) and Hedstrom files (Maillefer, Ballagius, Switzerland). Under a DOM (OPMI Pico; Carl Zeiss, Göttingen, Germany), two additional orifices, mesiolingual (ML) and mesiobuccal (MB), were identified around the previously treated distal (D) canal; however, only the D canal exhibited initial patency.

After two appointments, all three canals (MB, ML, and D) were negotiated to the working length, which was confirmed using an electronic apex locator (Root ZX; J Morita, Irvine, California, United States) and a radiograph. Two separate radiographs taken with a horizontal shift revealed that the files deviated from the center of the root, further indicating the potential existence of additional unnegotiated canals (Figure 1b).

We arranged a CBCT examination (Planmeca ProMax 3D Mid; Planmeca USA Inc., Roselle, Illinois, United States) for the patient. The axial view clearly indicated the presence of a fourth canal (Figure 2). It was determined that a distolingual (DL) canal had been missed, and the previously identified D canal was actually the distobuccal (DB) canal. Further microexploration was performed using the DOM, and patency to the working length of the DL canal was successfully achieved over the subsequent three appointments.

**Figure 2:**
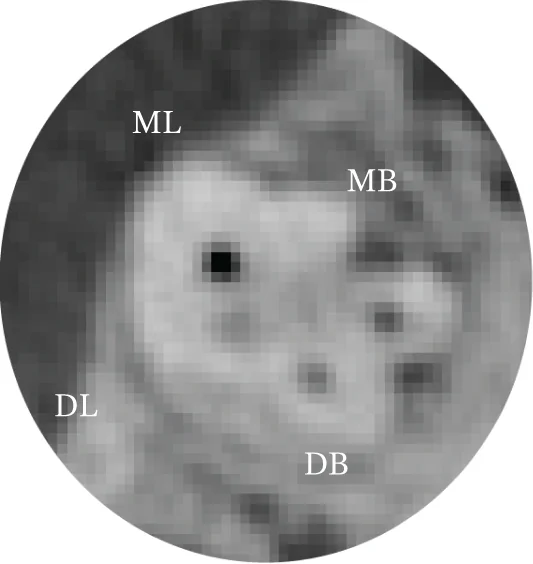
Cone‐beam computed tomography (CBCT) assessment. Axial slice of the CBCT scan confirming the presence of a fourth root canal in the mandibular right first premolar (Tooth 44). DB, distobuccal; DL, distolingual; MB, mesiobuccal; ML, mesiolingual.

Working lengths for all four canals were confirmed via the electronic apex locator and a radiograph (Figure 3a). The canals were mechanically prepared using rotary nickel‐titanium instruments (WaveOne gold Universal; Dentsply Maillefer, Ballaigues, Switzerland) up to 35.06%. Irrigation with 2.5% NaOCl was performed after each instrumentation change, and canal patency was maintained using a #10 K‐file (Maillefer, Ballaigures, Switzerland). Following preparation, calcium hydroxide paste (Daroupakhsh, Tehran, Iran) was placed in the canals as an intracanal medicament, and the access cavity was temporarily sealed with Cavit (GC Corporation, Tokyo, Japan).

**Figure 3:**
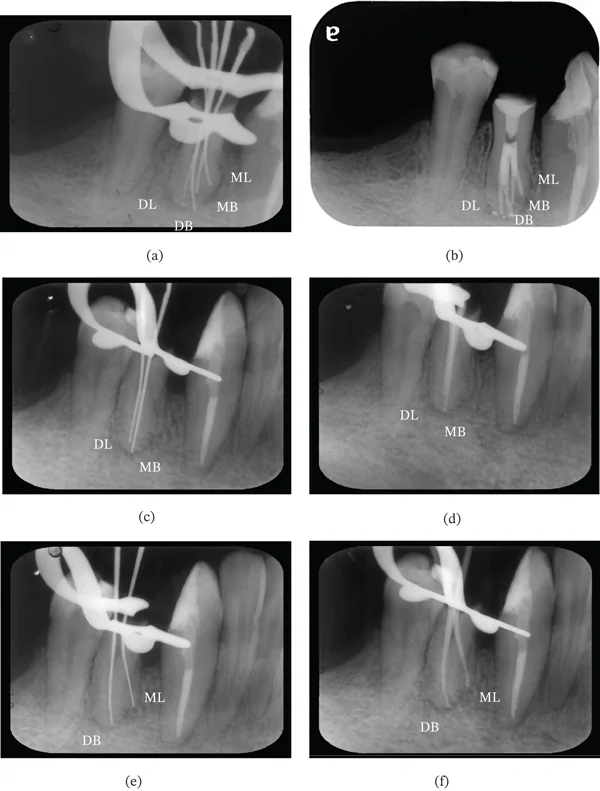
Endodontic treatment procedures and radiographic verification. (a) Working length determination radiograph confirmed using an electronic apex locator. (b) Postoperative radiograph demonstrating dense, complete three‐dimensional obturation of all four root canals. (c) Working length radiograph of the distolingual (DL) and mesiobuccal (MB) canals. (d) Master cone radiograph of the DL and MB canals. (e) Working length radiograph of the distobuccal (DB) and mesiolingual (ML) canals. (f) Master cone radiograph of the DB and ML canals.

One week later, the tooth remained completely asymptomatic. The canals were irrigated with 2.5% sodium hypochlorite, which was ultrasonically activated using an IRRI S 20/21 tip (Satelec Acteon, France) attached to Suprasson P5 unit. All canals were obturated using a warm vertical compaction technique with gutta‐percha (Gapadent, Hamburg, Germany) and AH plus sealer (Dentsply DeTrey, Konstanz, Germany) (Figure 3b). To prevent mutual interference, the master cones were placed on opposite sides in two separate stages, with a radiograph taken for verification after each placement. Following this, the DL and MB canals were obturated first, followed by the DB and ML canals (Figure 3c–f). The access cavity was temporarily sealed using Cavit and IRM (Dentsply Caulk, Milford, Delaware, United States), and the patient was referred for permanent restorative treatment.

An 8‐year follow‐up radiograph showed complete periapical healing; the full coronal coverage (PFM crown) remained stable, and the patient presented with no signs or symptoms. The tooth remained functional and asymptomatic (Figure 4).

**Figure 4:**
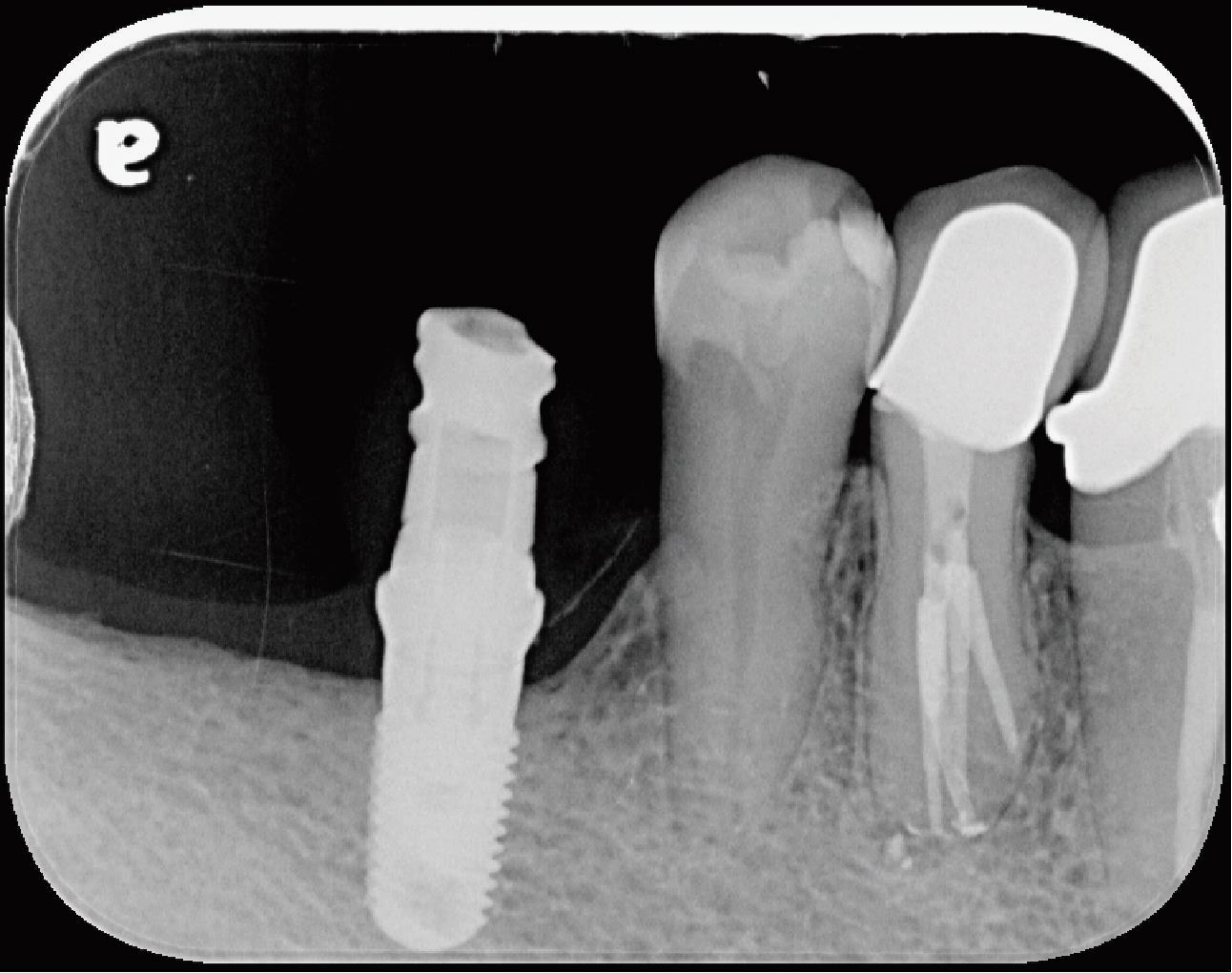
Postoperative follow‐up. An 8‐year follow‐up periapical radiograph demonstrating complete bone healing and the long‐term success of the endodontic treatment.

## 3. Discussion

According to Hoen and Pink [5], up to 42% of endodontic failures are attributed to missing canals, which can be significantly improved through endodontic retreatment. Slowey [6] demonstrated that mandibular premolars can be among the most difficult teeth to treat successfully due to their high anatomic variability. The complexity is largely influenced by confounding factors such as ethnicity, gender, and age. Therefore, clinicians must maintain a high index of suspicion regarding such anatomical variations.

In a systemic review, Cleghorn et al. [7] stated that although most mandibular first premolars present with a single root and a single canal, approximately 24.2% of cases exhibit two or more canals. Notably, the incidence of multiple roots or canals in mandibular first premolars is significantly higher in Chinese populations; two studies reported prevalences of 34.3% and 46% in Taiwanese cohorts [8, 9].

A recent review reported that the most common root canal configuration in mandibular first premolars is 1‐1‐1/1(70.6%), followed by 1‐1‐2/2 and 1‐2‐2/2(7.3% each) [10]. Ethnic variations also influence the prevalence of C‐shaped canal systems in mandibular first premolars, with Fan et al. [11] reporting an incidence of 24% in a Chinese population. Lu et al. [9] proposed the term “circumferential canals” to describe a morphology found in 6% of mandibular first premolars, characterized by a single central canal and three or four peripheral canals visible on the axial view. In the present case, the tooth exhibited a blunt root tip resembling circumferential canals; however, the canals divided into multiple distinct pathways in the middle section of the root, which are incompatible with typical circumferential canal features. Furthermore, the internal root canal system partially conforms to Type XI and Type XX of the Sert and Bayirli classification [12]; however, it does not completely fit any single established category (Figure 5), highlighting the limitation of current classification systems.

**Figure 5:**
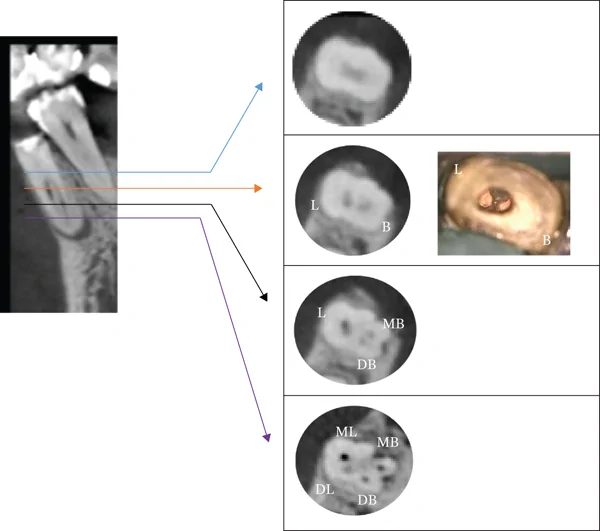
Serial CBCT axial views and postoperative microscopic appearance of Tooth 44. Sequential axial CBCT slices from the coronal to apical levels. The series demonstrates a transition from a single canal splitting into buccal and lingual pathways, followed by the division of the buccal canal into mesiobuccal and distobuccal branches, and the subsequent division of the lingual canal into mesiolingual and distolingual branches, finally resolving into four distinct root canals.

Although case reports documenting four or even five canals in mandibular first premolars exist [13–17], the presentation of four separate canals in a mandibular first premolar remains exceedingly rare.

With the advent of contemporary endodontic technologies, both the DOM and CBCT have greatly enhanced the clinician′s ability to locate hidden root canals. The illumination and magnification provided by the DOM offer a superior optical field to identify hidden orifices, remove canal obstructions, and detect structural defects such as cracks or fractures [18]. Furthermore, the DOM facilitates the precise detection and removal of remaining gutta‐percha and sealer remnants during retreatment [19]. Advanced 3D imaging techniques, such as CBCT, overcome the inherent limitations of conventional periapical radiography, which compresses three‐dimensional anatomy into a 2D plane. This 2D compression often obscures vital features of the tooth and its surrounding structures due to the superimposition of overlying tissues [20]. CBCT provides accurate 3D reconstruction of the teeth and maxillofacial bones with a lower radiation dose than conventional medical CT. High‐resolution CBCT imaging is considered an excellent tool for visualizing root canal morphology during preoperative assessment and diagnosis [21]. However, CBCT scans should not be prescribed routinely for standard endodontic treatments; they should be limited to cases where complex anatomy is suspected and conventional 2D imaging provides insufficient diagnostic clarity [22].

In the present case, the initial clue was the eccentric location of the previous root canal filling material, combined with the broadening of two distinct PDL spaces on the mesial aspect of the root. This radiographic evidence strongly suggested the presence of missing canals or accessory roots. Subsequently, shift periapical radiographs revealed that the endodontic files were eccentric within the root canal space. This radiographic finding strongly raised the suspicion of a missed canal, consistent with the diagnostic approach described by Jeong et al. for identifying complex canal configurations [23]. Consequently, a CBCT scan was performed, which definitively confirmed the presence of four distinct root canals.

## 4. Conclusion

This case report highlights a rare anatomical variation of a mandibular first premolar presenting with four root canals. The 8‐year follow‐up demonstrates not only complete periapical healing but also the long‐term functional stability and predictability of managing such complex aberrations. A thorough understanding of root canal morphology and a high index of suspicion for anatomical aberrations are critical for clinical success. Advanced magnification and imaging modalities, especially the DOM and CBCT, serve as invaluable adjuncts in diagnosing and managing complex endodontic cases. Ultimately, locating all canals, achieving meticulous three‐dimensional chemo‐mechanical preparation, and ensuring complete obturation—complemented by a high‐quality coronal restoration—remain the cornerstones of long‐term endodontic success [24, 25].

## Funding

No funding was received for this manuscript.

## Ethics Statement

This case report was conducted in accordance with the ethical standards of the Declaration of Helsinki. Institutional Review Board (IRB) approval was obtained from CGH (Approval No. [CGH‐P115096]). Written informed consent for publication of clinical details and images was obtained from the patient.

## Conflicts of Interest

The authors declare no conflicts of interest.

## Data Availability

The data that support the findings of this study are available on request from the corresponding author. The data are not publicly available due to privacy or ethical restrictions.
